# Effectiveness and Safety of Reduced Thymoglobulin Dosing in Low‐Risk Kidney Transplant Recipients

**DOI:** 10.1155/joot/4987172

**Published:** 2025-12-14

**Authors:** Eiman Wazwaz, Nina Joiner

**Affiliations:** ^1^ Department of Pharmacy, Cleveland Clinic Weston Hospital, Weston, Florida, USA

**Keywords:** antilymphocyte serum, graft rejection, immunosuppression, kidney transplantation, leukopenia, thrombocytopenia

## Abstract

**Introduction:**

Thymoglobulin, a lymphocyte‐depleting agent, is widely used for induction immunosuppression in kidney transplantation. Despite guideline support, there is no standardized dosing recommendation, resulting in variability across centers. In April 2022, our institution reduced its institutional practice thymoglobulin dose from 4.5 to 3 mg/kg for low‐risk kidney transplant recipients. This study aimed to evaluate the noninferiority of the reduced dose compared to the prior regimen in terms of effectiveness and safety.

**Methods:**

This single‐center retrospective noninferiority cohort study of low‐risk kidney transplant recipients was conducted from April 2020 to April 2024. Patients received either 3 or 4.5 mg/kg of thymoglobulin. The primary outcome was a composite of biopsy‐proven or suspected acute rejection, graft loss, or death within 6 months posttransplant. Secondary outcomes included leukopenia, thrombocytopenia, infections, delayed graft function, eGFR, malignancies, and hospital length of stay.

**Results:**

A total of 196 patients were included (116 in 4.5 mg/kg; 80 in 3 mg/kg). The primary outcome occurred in 11% and 3% of patients, respectively (risk difference −8.7%, 95% CI –15.4 to −2.0; *p* = 0.024). The reduced‐dose group experienced significantly lower rates of leukopenia, thrombocytopenia, and viral infections.

**Conclusion:**

A 3 mg/kg thymoglobulin dose is noninferior to 4.5 mg/kg and is associated with improved safety in low‐risk kidney transplant recipients.

## 1. Introduction

Kidney transplantation remains the preferred treatment for eligible patients with end‐stage renal disease (ESRD), offering superior survival and enhanced quality of life compared to maintenance dialysis [1, 2]. In the United States, the number of kidney transplants continues to rise, with the Organ Procurement and Transplantation Network (OPTN) reporting 27,759 kidney transplants performed in 2024 [3]. Despite advances in surgical techniques and maintenance immunosuppression, the incidence of acute rejection is highest during the early posttransplant period, highlighting the critical role of induction therapy [4].

Thymoglobulin, a rabbit‐derived polyclonal anti‐thymocyte globulin, is commonly employed as an induction agent to mitigate the risk of early allograft rejection [5]. Its immunosuppressive activity is mediated through depletion of CD4+ T lymphocytes and modulation of various immune pathways [6]. The pharmacodynamic effects of thymoglobulin are dose‐dependent, with rapid onset and prolonged duration, as lymphocyte recovery may extend up to 12 months postadministration [7, 8]. Current guidelines from Kidney Disease: Improving Global Outcomes (KDIGO) and the National Kidney Foundation (NKF) support the use of lymphocyte‐depleting agents such as thymoglobulin in patients with moderate to high immunologic risk; however, no consensus exists regarding a standardized dosing regimen [1, 2]. As a result, clinical practice varies widely, with cumulative thymoglobulin doses ranging from 3 to 10 mg/kg across transplant centers [9, 10].

Higher thymoglobulin cumulative doses have been linked to an increased risk of adverse effects, including leukopenia, thrombocytopenia, infections, and malignancies [11–13]. These complications are particularly concerning in low‐risk transplant recipients, who may receive little additional benefit from higher levels of immunosuppression [14]. Nevertheless, many transplant centers have historically administered fixed thymoglobulin doses irrespective of immunologic risk.

In April 2022, our transplant center implemented a risk‐stratified induction protocol, reducing the cumulative thymoglobulin dose to 3 mg/kg for low‐risk kidney transplant recipients while maintaining the 4.5 mg/kg regimen for high‐risk patients. Given the limited evidence directly comparing these dosing strategies in risk‐stratified cohorts, this study aims to evaluate the effectiveness and safety of a reduced 3 mg/kg thymoglobulin dose compared to 4.5 mg/kg in low‐risk kidney transplant recipients. This study aims to optimize induction therapy in low‐risk recipients by minimizing adverse effects associated with over‐immunosuppression while preserving clinical efficacy.

## 2. Materials and Methods

This retrospective, single‐center, observational noninferiority cohort study included adult kidney transplant recipients who received thymoglobulin induction therapy between April 2020 and April 2024. Patients were included if they met predefined criteria for low immunologic risk, defined as age ≥ 50 years, no prior organ transplantation, and a calculated panel reactive antibody (cPRA) < 20%. In the absence of a universally accepted definition, low risk was defined using criteria based on multiple factors reported in the literature to influence rejection risk and consistent with the approach at the institution where the study took place. Patients were excluded if they did not meet all low‐risk eligibility criteria, received basiliximab as the induction agent, or underwent multiorgan transplantation.

Prior to April 2022, all kidney transplant recipients received a fixed cumulative dose of thymoglobulin 4.5 mg/kg during their transplant hospitalization. In April 2022, the institution implemented a risk‐stratified protocol in which patients meeting low‐risk criteria received a reduced cumulative dose of 3 mg/kg. Patients were stratified into two groups based on the total cumulative dose of thymoglobulin administered during the index transplant admission. Low‐risk patients who received 4.5 mg/kg before April 2022 were compared with those who received 3 mg/kg after implementation of the risk‐stratified protocol. Low‐risk patients administered a higher dose after the protocol change at clinician discretion were analyzed in the high‐dose group.

All kidney transplant recipients received the institution’s maintenance immunosuppression protocol, which consisted of tacrolimus, mycophenolate mofetil, and prednisone. Tacrolimus dosing was adjusted according to trough concentrations, mycophenolate mofetil was administered at 1000 mg twice daily, and prednisone was tapered to 5 mg daily. Antiviral prophylaxis was prescribed based on donor and recipient serologic status.

All outcomes were assessed over a 6‐month follow‐up period posttransplantation. The primary outcome was a composite of biopsy‐proven acute rejection (BPAR), suspected acute rejection, graft loss, and patient death. BPAR was defined as histologically confirmed rejection on renal allograft biopsy. Suspected rejection was defined as the initiation of anti‐rejection therapy based on clinical judgment in the absence of biopsy confirmation. All biopsies were indication‐based and performed for unexplained graft dysfunction, such as elevated serum creatinine, proteinuria, or other concerning findings identified by the treating clinician. Graft loss was defined as permanent loss of allograft function requiring dialysis, and patient death was defined as all‐cause mortality during the follow‐up period.

**Figure 1:**
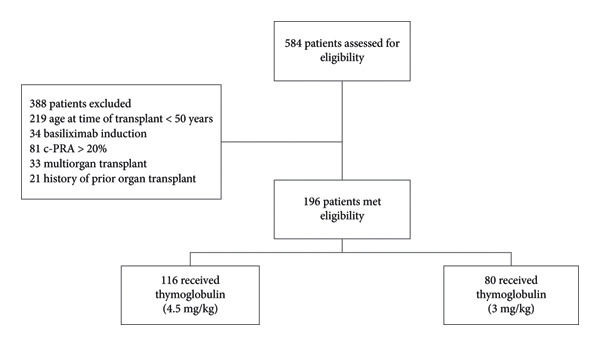
Flowchart of patients.

Secondary outcomes included the incidence of leukopenia, defined as white blood cell count less than 4.0 × 10^3^/μL; thrombocytopenia, defined as platelet count less than 150 × 10^3^/μL; culture‐proven bacterial, fungal, and viral infections, with viral infections including CMV and BK viremia to capture opportunistic pathogens; delayed graft function; estimated glomerular filtration rate (eGFR); new‐onset or recurrent malignancies; and hospital length of stay. The flow of patient screening, exclusions, and group assignment is detailed in Figure 1.

Sample size estimation was based on a noninferiority design to compare the composite primary outcome between the two thymoglobulin dosing groups. Assuming a 10% event rate in both groups, a noninferiority margin of 5%, a one‐sided alpha of 0.05%, and 80% power, a total of 892 patients (446 per group) were required to demonstrate noninferiority with adequate statistical power. Descriptive statistics were used to summarize baseline characteristics and outcomes. Continuous variables were expressed as means with standard deviations (SDs) or medians with interquartile ranges (IQRs), as appropriate. Categorical variables were summarized as frequencies and percentages. Comparisons between groups were conducted using Student’s *t*‐test or Mann–Whitney *U* test for continuous variables, and chi‐square or Fisher’s exact test for categorical variables. Noninferiority of the 3 mg/kg dose relative to 4.5 mg/kg was assessed by evaluating the absolute risk difference in the composite primary outcome along with the corresponding two‐sided 95% confidence interval. Noninferiority was concluded if the upper limit of the confidence interval did not exceed the prespecified noninferiority margin.

All statistical analyses were conducted using Stata software (Version 19; StataCorp, College Station, TX), and a *p* value < 0.05 was considered statistically significant. Institutional review board approval was obtained prior to study initiation.

## 3. Results

A total of 196 kidney transplant recipients were included in the study, with 116 patients receiving thymoglobulin 4.5 mg/kg and 80 patients receiving 3 mg/kg (Table 1). Baseline characteristics were well balanced between groups; therefore, multivariate adjustment for potential confounders was not performed. The median recipient age was 63 years in the 4.5 mg/kg group and 65 years in the 3 mg/kg group. The cohort was predominantly male (73%), with similar gender distribution across groups. The racial distribution was also comparable, with Black and White recipients comprising the majority (41% and 46%, respectively), followed by multiracial (10%) and Asian (3%) individuals. Immunologic risk was low in both groups; 89% of patients had a cPRA < 10%. The median number of HLA mismatches was 4 in the 4.5 mg/kg group and 5 in the 3 mg/kg group.

**Table 1:** Baseline characteristics.

Variable	Total *n* = 196	Thymoglobulin 4.5 mg/kg group *n* = 116	Thymoglobulin 3 mg/kg group *n* = 80	Statistical significance (*p* value)
Recipient age^1^	63 (58–69)	63 (58–69)	65 (59–72)	0.163
Recipient gender, male	144 (73)	87 (75)	57 (71)	0.559
Recipient race				0.353
White = 1	91 (46)	50 (43)	41 (51)	
Black = 2	80 (41)	53 (46)	27 (34)	
Asian = 3	5 (3)	2 (2)	3 (4)	
Multiracial = 4	20 (10)	11 (10)	9 (11)	
Recipient weight^2^ (kg)	81 ± 17	88 ± 16	86 ± 19	0.518
Recipient BMI	29 ± 5	30 ± 5	29 ± 5	0.462
Duration on dialysis^1^ (months)	54 (23–75)	51 (24–73)	58 (22–78)	0.587
c‐PRA %				0.721
0	174 (89)	101 (87)	73 (91)	
1–9	15 (8)	10 (9)	5 (6)	
10–20	7 (4)	5 (4)	2 (3)	
HLA mismatch^1^	4 (4–5)	4 (4–5)	5 (3–5)	0.829
Thymoglobulin cumulative dose^1^ (mg)	300 (263–438)	375 (313–450)	250 (200–300)	< 0.001
Thymoglobulin cumulative dose^1^ (mg/kg)	4.0 (3.1–4.4)	4.4 (4.1–4.7)	3.0 (2.7–3.3)	< 0.001
Donor age^1^	46 (37–57)	46 (37–58)	46 (36–55)	0.615
Donor status, deceased	166 (85)	96 (83)	70 (88)	0.365
Deceased donor cause of death				0.474
DBD	121 (73)	72 (75)	49 (70)	
DCD	45 (27)	24 (25)	21 (30)	
Cold ischemia time^1^ (minutes)	1137 (930–1332)	1113 (880–1327)	1219 (979–1343)	0.101
Recipient CMV status, positive	144 (73)	81 (70)	63 (79)	0.164
Donor CMV status, positive	125 (64)	71 (61)	54 (68)	0.368
Recipient comorbidities				
DM	89 (45)	53 (46)	36 (45)	0.924
HTN	186 (95)	111 (96)	75 (94)	0.544
CHF	6 (3)	2 (2)	4 (5)	0.228
History of malignancy	32 (16)	20 (17)	12 (15)	0.676
ESRD etiology				
GN	31 (16)	22 (19)	9 (11)	0.146
DM	79 (40)	48 (41)	31 (39)	0.712
HTN	125 (64)	71 (61)	54 (68)	0.368
OU	3 (2)	1 (1)	2 (3)	0.568
PKD	12 (6)	6 (5)	6 (8)	0.553
Other	15 (8)	9 (8)	6 (8)	0.947

*Note:* Characteristics are listed as no. (%) unless otherwise noted.

Abbreviations: BMI, body mass index; CHF, congestive heart failure; CMV, cytomegalovirus; cPRA, calculated panel reactive antibodies; DBD, donation after brain death; DCD, donation after circulatory death; DM, diabetes mellitus; ESRD, end‐stage renal disease; GN, glomerulonephritis; HLA, human leukocyte antigen; HTN, hypertension; IQR, interquartile range; OU, obstructive uropathy; PKD, polycystic kidney disease; SD, standard deviation.

^1^Median (IQR).

^2^Mean ± SD.

Common comorbidities included hypertension (95%), diabetes mellitus (45%), prior malignancy (16%), and congestive heart failure (3%), with no significant differences between groups. As expected, the cumulative thymoglobulin dose differed by group: the 4.5 mg/kg group received a median dose of 4.4 mg/kg (IQR, 4.1–4.7), while the 3 mg/kg group received 3.0 mg/kg (IQR, 2.7–3.3).

**Figure 2:**
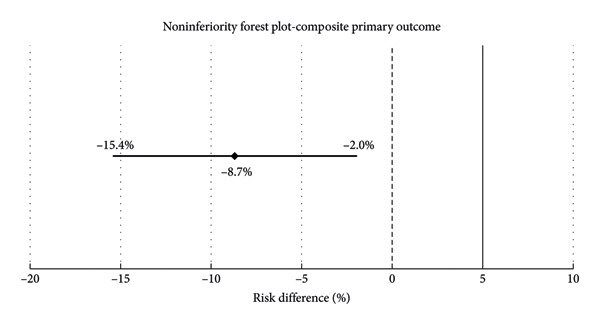
Noninferiority forest plot for the composite primary outcome.

The composite primary outcome, evaluated over six months posttransplant, occurred in 15 patients (8%) overall. This included 13 patients (11%) in the 4.5 mg/kg group and 2 patients (3%) in the 3 mg/kg group (*p* = 0.024). The absolute risk difference was −8.7% (95% CI, −15.4% to −2.0%), and the entire confidence interval remained below the prespecified noninferiority margin of 5%, confirming noninferiority of the 3 mg/kg dose compared to the 4.5 mg/kg dose (Figure 2).

Individually, BPAR occurred in 1 patient (1%) in the 4.5 mg/kg group and in none of the patients in the 3 mg/kg group (*p* = 1.000). Suspected acute rejection was observed in 2 patients (2%) in the 4.5 mg/kg group and none in the 3 mg/kg group (*p* = 0.529). Graft loss occurred in 7 patients (6.0%) in the 4.5 mg/kg group and in none of the patients in the 3 mg/kg group (*p* = 0.089). Patient death occurred in 3 patients (3%) in the 4.5 mg/kg group and in 2 patients (3%) in the 3 mg/kg group (*p* = 1.000) (Table 2).

**Table 2:** Primary outcomes at 6 months.

Variable	Total *n* = 196	Thymoglobulin 4.5 mg/kg group *n* = 116	Thymoglobulin 3 mg/kg group *n* = 80	Statistical significance
Composite primary Outcomes	15 (8)	13 (11)	2 (3)	*p* = 0.024 Risk difference: −8.7% 95% CI: (−15.4 to −2.0)
BPAR	1 (0.5)	1 (1)	0 (0)	1.000
Suspected acute rejection	2 (1)	2 (2)	0 (0)	0.529
Graft loss	7 (4)	7 (6)	0 (0)	0.089
Patient death	5 (3)	3 (3)	2 (3)	1.000

*Note:* Outcomes are listed as no. (%) unless otherwise noted.

Abbreviation: BPAR, biopsy‐proven acute rejection.

Leukopenia occurred in 94 patients (81%) in the 4.5 mg/kg group and in 55 patients (69%) in the 3 mg/kg group (*p* = 0.048). Thrombocytopenia was reported in 101 patients (87%) in the 4.5 mg/kg group and in 55 patients (69%) in the 3 mg/kg group (*p* = 0.002). Viral infections occurred in 82 patients (71%) in the 4.5 mg/kg group and in 42 patients (53%) in the 3 mg/kg group (*p* = 0.009). Bacterial infections were observed in 62 patients (53%) in the 4.5 mg/kg group and in 40 patients (50%) in the 3 mg/kg group. Fungal infections occurred in 6 patients (5%) and 4 patients (5%) in the 4.5 and 3 mg/kg groups, respectively.

New malignancies were identified in 4 patients (3%) in the 4.5 mg/kg group and in 1 patient (1%) in the 3 mg/kg group. Recurrent malignancy occurred in 1 patient (1%) in the 4.5 mg/kg group and was not observed in the 3 mg/kg group. The median eGFR at follow‐up was 52 mL/min/1.73 m^2^ (IQR, 42–60) in the 4.5 mg/kg group and 53 mL/min/1.73 m^2^ (IQR, 44–72) in the 3 mg/kg group (*p* = 0.053). Delayed graft function occurred in 38 patients (33%) in the 4.5 mg/kg group and in 21 patients (26%) in the 3 mg/kg group. The median hospital length of stay was 4 days (IQR, 3–5) in the 4.5 mg/kg group and 4.5 days (IQR, 3–6) in the 3 mg/kg group (Table 3).

**Table 3:** Secondary outcomes at 6 months.

Variable	Total *n* = 196	Thymoglobulin 4.5 mg/kg group *n* = 116	Thymoglobulin 3 mg/kg group *n* = 80	Statistical significance (*p* value)
eGFR^1^ (mL/min/1.73 m^2^)	52 (43–61)	52 (42–60)	53 (44–72)	0.053
DGF	59 (30)	38 (33)	21 (26)	0.329
Leukopenia	149 (76)	94 (81)	55 (69)	0.048
Thrombocytopenia	156 (80)	101 (87)	55 (69)	0.002
Bacterial infections	102 (52)	62 (53)	40 (50)	0.635
Viral infections	124 (63)	82 (71)	42 (53)	0.009
Fungal infections	10 (5)	6 (5)	4 (5)	1.000
New malignancy	5 (3)	4 (3)	1 (1)	0.650
Recurrent malignancy	1 (1)	1 (1)	0 (0)	1.000
Hospital length of stay^1^	4 (3–6)	4 (3–5)	4.5 (3–6)	0.481

*Note:* Outcomes are listed as no. (%) unless otherwise noted.

Abbreviations: DGF, delayed graft function; eGFR, estimated glomerular filtration rate; IQR, interquartile range.

^1^Median (IQR).

## 4. Discussion

In this single‐center, retrospective noninferiority study of low‐risk kidney transplant recipients, a reduced thymoglobulin induction dose of 3 mg/kg was noninferior to the standard 4.5 mg/kg dose in preventing acute rejection, graft loss, or death at 6 months posttransplant. The incidence of the composite primary outcome was significantly lower in the 3 mg/kg group compared to the 4.5 mg/kg group (3% vs. 11%), with an absolute risk difference of −8.7% (95% CI, −15.4% to −2.0%), satisfying the prespecified noninferiority criterion. Biopsy‐proven and suspected acute rejection rates were low across both dosing strategies. However, rejection was numerically higher in the 4.5 mg/kg group, representing a paradoxical finding, as higher thymoglobulin exposure would typically be expected to reduce rejection risk. Variations in maintenance immunosuppression exposure, tacrolimus trough levels, and patient adherence may also have influenced outcomes, but these factors were not captured in this study.

While graft loss and mortality occurred less frequently in the reduced‐dose group, these differences were not statistically significant. Among the 4.5 mg/kg group, one patient died from cardiac arrest with suspected COVID‐19–related complications following delayed graft function and postoperative dialysis. Another patient died from cardiac arrest following ventricular fibrillation and subsequently developed multiorgan failure. The remaining deaths in this group had undocumented causes. In the 3 mg/kg group, one patient died from invasive nasopharyngeal mucormycosis with sepsis and cardiac arrest; the postresuscitation course was complicated by myoclonic seizures and suspected anoxic brain injury. The second death had no documented cause. These findings support the use of low‐dose thymoglobulin as a viable induction strategy in low‐risk recipients without evidence of increased mortality.

The reduced‐dose group experienced significantly lower rates of leukopenia, thrombocytopenia, and viral infections. These adverse events are commonly linked to excessive lymphocyte depletion and can compromise posttransplant recovery or limit the ability to maintain standard maintenance immunosuppression. In this analysis, viral infections included CMV and BK viremia, highlighting a reduction in opportunistic viral infections within this immunocompromised population. The improved hematologic and infectious safety profile observed in the 3 mg/kg group reinforces the clinical value of minimizing thymoglobulin exposure when immunologic risk permits. It should be recognized that other medications routinely administered after kidney transplantation can also contribute to cytopenias, and their potential impact was not evaluated in this study. Renal function at 6 months, as measured by eGFR, was numerically higher in the reduced‐dose group, and delayed graft function occurred less frequently, although these differences did not reach statistical significance. Hospital length of stay was slightly longer in the reduced‐dose cohort, a counterintuitive finding given that prolonged hospitalization would be expected in the high‐dose group due to the additional infusion day. As data on potential contributors to length of stay, including postsurgical complications and socioeconomic factors, were not collected, the basis for this difference remains unclear. Overall, hospital stay was comparable between groups, suggesting that reduced induction dosing did not delay early postoperative recovery.

These findings are consistent with previous studies investigating lower thymoglobulin dosing in kidney transplantation. Laftavi et al. reported improved long‐term graft outcomes and reduced rejection rates with low‐dose thymoglobulin (3–5 mg/kg) in deceased donor recipients, without increased infectious or malignant complications [15]. Similarly, Lee et al. found that a 4.5 mg/kg regimen given over 3 days resulted in fewer biopsy‐proven rejection episodes in low‐risk patients [16]. Martinez‐Mier et al. evaluated a 3 mg/kg regimen in living donor recipients and demonstrated comparable safety and efficacy to standard‐dose induction [17], and Nafar et al. showed that a 4.5 mg/kg dose delivered over 3 days was associated with fewer complications and shorter hospital stays than either single‐dose or high‐dose protocols [18]. While prior studies have examined a range of dosing strategies, few have directly compared 3 and 4.5 mg/kg in a homogenous low‐risk cohort. The present study addresses this gap and provides real‐world evidence that 3 mg/kg is not only noninferior for early graft outcomes but also offers an improved safety profile with fewer hematologic and viral events.

Several limitations should be acknowledged. As a single‐center retrospective analysis, the study is subject to inherent biases, including unmeasured confounding and limited generalizability to other settings with different patient populations or immunosuppressive practices. The study did not meet its originally planned sample size, which may have reduced power to detect differences in individual outcomes. The 6‐month follow‐up period reflects only early posttransplant outcomes and does not permit assessment of long‐term risks such as chronic rejection, malignancy, or graft survival. The impact of concomitant medications associated with hematologic cytopenias was not assessed. Important risk factors for acute rejection, such as donor‐specific antibody status, cell‐free DNA levels, rejection classification, maintenance immunosuppressive exposure, tacrolimus monitoring, and patient adherence, were also not evaluated and may have influenced rejection outcomes. Future studies incorporating longer follow‐up and a more comprehensive set of clinical and immunologic variables are needed to confirm the durability and safety of reduced‐dose thymoglobulin.

## 5. Conclusion

This retrospective, single‐center noninferiority study demonstrates that a reduced thymoglobulin induction dose of 3 mg/kg is noninferior to the standard 4.5 mg/kg dose in preventing acute rejection, graft loss, or death at 6 months in low‐risk kidney transplant recipients. The low‐dose regimen was associated with fewer cytopenias and viral infections and did not adversely affect graft function or early recovery.

These findings support reduced‐dose thymoglobulin as a safe, effective, and cost‐conscious induction strategy in appropriately selected patients. Prospective multicenter studies are needed to confirm these results and to inform the development of individualized, risk‐based dosing models for optimizing induction immunosuppression in kidney transplantation.

**Nomenclature**
IRB: Institutional Review Board
eGFR: Estimated glomerular filtration rate
CI: Confidence interval

## Disclosure

No financial support or sponsorship influenced any aspect of the study.

## Conflicts of Interest

The authors declare no conflicts of interest.

## Funding

This study was completed without external funding. The research was internally supported through routine institutional resources at Cleveland Clinic Weston Hospital.

## Data Availability

The data that support the findings of this study are available from the corresponding author upon reasonable request.
